# A pilot cohort study of cerebral autoregulation and 2-year neurodevelopmental outcomes in neonates with hypoxic-ischemic encephalopathy who received therapeutic hypothermia

**DOI:** 10.1186/s12883-015-0464-4

**Published:** 2015-10-20

**Authors:** Vera Joanna Burton, Gwendolyn Gerner, Elizabeth Cristofalo, Shang-en Chung, Jacky M. Jennings, Charlamaine Parkinson, Raymond C. Koehler, Raul Chavez-Valdez, Michael V. Johnston, Frances J. Northington, Jennifer K. Lee

**Affiliations:** Neurology and Developmental Medicine, Kennedy Krieger Institute, Baltimore, MD USA; Neurosciences Intensive Care Nursery, Johns Hopkins School of Medicine, Baltimore, MD USA; Department of Neurology, Johns Hopkins School of Medicine, Baltimore, MD USA; Department of Neuropsychology, Kennedy Krieger Institute, Baltimore, MD USA; Division of Perinatal-Neonatal Medicine, Department of Pediatrics, Johns Hopkins School of Medicine, Baltimore, MD USA; Center for Child and Community Health Research (CCHR), Department of Pediatrics, Johns Hopkins School of Medicine, Baltimore, MD USA; Department of Anesthesiology and Critical Care Medicine, Johns Hopkins School of Medicine, Baltimore, MD USA; Hugo Moser Research Institute, Kennedy Krieger Institute, Baltimore, MD USA; Department of Neurology and Developmental Medicine, Kennedy Krieger Institute, Johns Hopkins School of Medicine, 801 N Broadway, Baltimore, MD 21205 USA

**Keywords:** Autoregulation, NIRS, Hypoxic-Ischemic Encephalopathy, Therapeutic Hypothermia, Neurodevelopmental Outcomes

## Abstract

**Background:**

Neurodevelopmental disabilities persist in survivors of neonatal hypoxic-ischemic encephalopathy (HIE) despite treatment with therapeutic hypothermia. Cerebrovascular autoregulation, the mechanism that maintains cerebral perfusion during changes in blood pressure, may influence outcomes. Our objective was to describe the relationship between acute autoregulatory vasoreactivity during treatment and neurodevelopmental outcomes at 2 years of age.

**Methods:**

In a pilot study of 28 neonates with HIE, we measured cerebral autoregulatory vasoreactivity with the hemoglobin volume index (HVx) during therapeutic hypothermia, rewarming, and the first 6 h of normothermia. The HVx, which is derived from near-infrared spectroscopy, was used to identify the individual optimal mean arterial blood pressure (MAP_OPT_) at which autoregulatory vasoreactivity is greatest. Cognitive and motor neurodevelopmental evaluations were completed in 19 children at 21–32 months of age. MAP_OPT_, blood pressure in relation to MAP_OPT_, blood pressure below gestational age + 5 (ga + 5), and regional cerebral oximetry (rSO_2_) were compared to the neurodevelopmental outcomes.

**Results:**

Nineteen children who had HIE and were treated with therapeutic hypothermia performed in the average range on cognitive and motor evaluations at 21–32 months of age, although the mean performance was lower than that of published normative samples. Children with impairments at the 2-year evaluation had higher MAP_OPT_ values, spent more time with blood pressure below MAP_OPT_, and had greater blood pressure deviation below MAP_OPT_ during rewarming in the neonatal period than those without impairments. Greater blood pressure deviation above MAP_OPT_ during rewarming was associated with less disability and higher cognitive scores. No association was observed between rSO_2_ or blood pressure below ga + 5 and neurodevelopmental outcomes.

**Conclusion:**

In this pilot cohort, motor and cognitive impairments at 21–32 months of age were associated with greater blood pressure deviation below MAP_OPT_ during rewarming following therapeutic hypothermia, but not with rSO_2_ or blood pressure below ga + 5. This suggests that identifying individual neonates’ MAP_OPT_ is superior to using hemodynamic goals based on gestational age or rSO_2_ in the acute management of neonatal HIE.

**Electronic supplementary material:**

The online version of this article (doi:10.1186/s12883-015-0464-4) contains supplementary material, which is available to authorized users.

## Background

Neonatal hypoxic-ischemic encephalopathy (HIE) affects approximately 3 in 1000 births and is the most common cause of perinatal brain injury in full-term neonates [1, 2]. Long-term severe sequelae of neonatal HIE include intellectual disability and cerebral palsy. In children who received therapeutic hypothermia for HIE, the incidence of cerebral palsy is approximately 17 % and the incidence of IQ < 70 is 27 % [3]. Based on these incidence rates, in the United States, the financial burden of HIE-induced intellectual disabilities exceeds $3.4 billion per year, and the costs of HIE-induced cerebral palsy exceed $1.9 billion per year [3–5]. Multicenter, randomized controlled trials of therapeutic hypothermia for neonatal HIE demonstrate incomplete neuroprotection. In the Total Body Hypothermia for Neonatal Encephalopathy Trial, 55 % of HIE survivors who received hypothermia had persistent neurologic abnormalities at age 6–7 years, including 21 % with cerebral palsy and 22 % with moderate or severe disabilities [6]. The National Institute of Child Health and Human Development (NICHD) Neonatal Research Network trial of therapeutic hypothermia in HIE found that 35 % of survivors who received hypothermia had moderate or severe disabilities at 6–7 years of age [3]. Therefore, additional modifiable factors and potential adjuvant therapies to hypothermia must be identified to improve neurologic outcomes.

Dysregulated cerebral blood flow may be a key component in secondary neurologic injury in HIE [7]. Cerebrovascular autoregulation maintains relatively constant cerebral blood flow across changes in perfusion pressure. This physiologic mechanism functions within a specific range of blood pressure, and the mean arterial blood pressure (MAP) with optimal autoregulatory function is termed the optimal MAP (MAP_OPT_). The hemoglobin volume index (HVx) monitors autoregulatory vasoreactivity by correlating changes in arterial blood pressure to changes in relative total tissue hemoglobin (rTHb), a surrogate measure of cerebral blood volume obtained by near-infrared spectroscopy (NIRS). HVx is based on the premise that autoregulatory vasodilation and vasoconstriction induce changes in cerebral blood volume that are proportional to changes in rTHb [8]. HVx can identify MAP_OPT_ in neonates with HIE [9, 10]. We previously reported that blood pressure deviation below MAP_OPT_ during rewarming is associated with greater brain injury on MRI in pilot studies of autoregulation during HIE [9, 10]. However, whether blood pressure autoregulation during therapeutic hypothermia and rewarming in the neonatal period is associated with later neurodevelopmental outcomes remains unknown. Neuroprotective blood pressure ranges for HIE are poorly defined, and many clinicians use regional cerebral oximetry (rSO_2_) or maintain blood pressures at gestational age in weeks +5 mmHg (ga + 5) to help guide hemodynamic goals in neonates [11].

The goal of this observational pilot study was to describe the relationship between blood pressure autoregulation during therapeutic hypothermia for treatment of neonatal HIE and cognitive and motor neurodevelopmental outcomes at approximately 2 years of age. We hypothesized that 1) greater blood pressure deviation below MAP_OPT_ would be associated with neurodevelopmental disabilities; 2) the rSO_2_ would not be associated with disability; and 3) greater time spent with blood pressure below the ga + 5 would not be associated with disability. We tested each of these hypotheses by comparing autoregulation measurements made during hypothermia, rewarming, and the first 6 h of normothermia to neurodevelopmental outcomes of children 2 years later.

## Methods

This study was approved by the Johns Hopkins University (JHU, Baltimore, MD) Institutional Review Board. Written, informed consent for HVx monitoring was obtained from the neonates’ parents upon admission to the JHU neonatal intensive care unit (NICU) and again before the 2-year neurodevelopmental follow-up evaluations, which took place at the Kennedy Krieger Institute (KKI, Baltimore, MD). All neonates who were admitted to the NICU between September 2010 and October 2012 were screened for study eligibility, which was based on the diagnosis of HIE according to criteria used by the NICHD Neonatal Research Network’s clinical trial of hypothermia in neonatal HIE [12]. Briefly, these infants were diagnosed with moderate to severe HIE based on clinical exam and blood gas from the umbilical cord or first hour of life with pH <7.15 or a base deficit >10 mmol/L. If a blood gas measurement was not available, 10-min Apgar score <5 or assisted ventilation for ≥10 min after birth, an acute perinatal event, and moderate to severe encephalopathy were used to diagnose HIE. Additional eligibility criteria for this pilot study included gestational age ≥35 weeks, birth weight ≥1800 g, initiation of whole-body cooling within 6 h of birth, presence of an arterial blood pressure cannula, and a parent who spoke English as the primary language. Neonates who did not have an arterial blood pressure cannula, who had a coagulopathy with active bleeding, or who had congenital anomalies or other diagnoses that could make cooling unsafe were not eligible for the study. Moreover, children who were involved in the foster care system at the time of neurodevelopmental follow-up were ineligible for the study. Seventeen of the children in the current study were part of the cohort in which we previously reported an association between blood pressure autoregulatory vasoreactivity measured by HVx and brain injuries on MRI [9].

### Clinical care in the NICU

All clinical care was determined by the treating clinicians and by NICU protocol. Neonates received whole-body hypothermia with a cooling blanket (Mul-T-Blanket Hyper/Hypothermia Blanket and Mul-T-Pad Temperature Therapy Pad; Gaymar Medi-Therm III, Gaymar Industries, Orchard Park, NY) to a goal rectal temperature of 33.5 ± 0.5 °C for 72 h. They were rewarmed over 6 h (goal 0.5 °C/h) to normothermia (36.5 °C). The clinicians determined the hemodynamic goals, decided when to implement vasoactive or inotropic medications, and selected the sedation regimens. When vasoactive medications were needed, dopamine was initiated followed by dobutamine, epinephrine, or milrinone infusions as necessary. Morphine, fentanyl, or hydromorphone boluses and infusions were used for sedation as necessary. Full montage electroencephalograms (EEGs) were conducted during hypothermia and after rewarming in addition to continuous amplitude-integrated EEG monitoring (Brainz BRM3 Monitor or CFM Olympic Brainz Monitor, Natus Medical Inc., San Carlos, CA) during hypothermia, rewarming, and the first 6 h of normothermia. Phenobarbital was administered to treat electrographic or clinical seizures; thereafter, levetiracetam, fosphenytoin, or topiramate was used for persistent seizures. Clinicians could view the rSO_2_, as measured by the NIRS, and blood pressure, as measured by continuous cardio-respiratory monitors, but they were blinded to HVx. Respiratory support parameters, including nasal cannula, high flow nasal cannula, or ventilator support with endotracheal tube were recorded during the rewarming period. Clinical histories and clinical variables were obtained by chart reviews.

### Autoregulation monitoring

Adhesive, neonatal cerebral oximetry probes were placed bilaterally on the neonates’ foreheads and connected to an INVOS 5100 NIRS machine (INVOS; Covidien, Boulder, CO) according to manufacturer guidelines. We synchronously sampled the NIRS signals and arterial blood pressure from the patient hemodynamic monitor at 100 Hz and processed the data with ICM+ software (Cambridge Enterprises, Cambridge, UK) using a bedside computer. The ICM+ software calculated HVx using a continuous, moving correlation coefficient between MAP and the rTHb (a surrogate measure of cerebral blood volume obtained by NIRS) after filtering out high-frequency waves from pulse and respiration [8, 13]. Each calculation of HVx incorporated consecutive, paired, 10-s averaged values from 300-s duration, thereby utilizing 30 data points for each HVx calculation. HVx is a continuous variable that ranges from –1 to +1. Negative or near-zero HVx represents functional vasoreactivity (and therefore intact autoregulation) because MAP and rTHb either negatively correlate or are not correlated. When blood pressure decreases and vasoreactivity becomes impaired, HVx becomes positive and approaches +1 because MAP and rTHb positively correlate. We manually removed artifacts in the NIRS and MAP signals (e.g., arterial line flushes), and we excluded data that comprised <1 % of the recording period as an additional measure to remove artifacts.

**Fig. 1:**
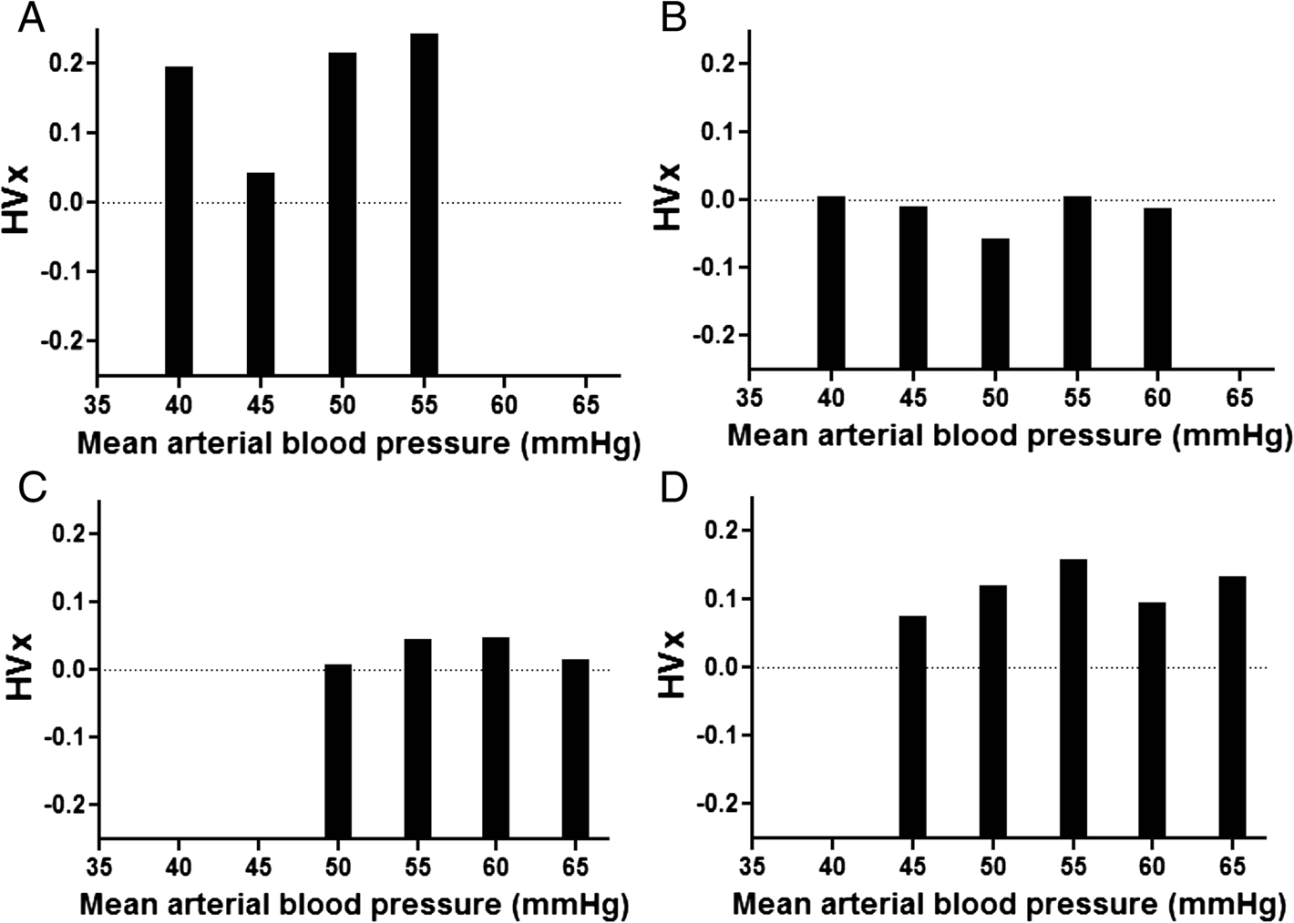
Representative hemoglobin volume index (HVx) bar graphs from individual neonates illustrate identification of the optimal mean arterial blood pressure (MAP_OPT_) at the nadir of HVx. MAP_OPT_ values were 45 mmHg for patient 1 (**a**) and 50 mmHg for patient 2 (**b**). Patients 5 (**c**) and 25 (**d**) did not have a nadir in HVx and were therefore coded as having an unidentifiable MAP_OPT_

The right and left HVx values were averaged and sorted into 5-mmHg bins of MAP to generate bar graphs. (None of the neonates had unilateral intracranial lesions on follow-up MRI.) We identified the MAP_OPT_ in each time period (hypothermia, rewarming, first 6 h of normothermia) as the bin that had the most negative HVx when the bar graph exhibited an overall trend of increasing HVx values as MAP deviated from this nadir (Fig. 1a and b). When a nadir in HVx could not be identified, the neonate was coded as having an unidentifiable MAP_OPT_ (Fig. 1c and d). These values were identified by an investigator who was blinded to the neurodevelopmental outcome (JKL).

Blood pressure data were analyzed by three methods within each of the three time periods. First, we calculated the amount of time the neonate spent with blood pressure below, at, or above MAP_OPT_ and analyzed this as a percentage of the autoregulation monitoring period. Second, we determined the maximal blood pressure deviation below or above MAP_OPT_ [9, 10]. Third, we calculated the area under the curve (AUC) to combine the extent of blood pressure deviation below MAP_OPT_ and the amount of time spent with blood pressure below MAP_OPT_. We analyzed time as the absolute duration of autoregulation monitoring to determine the AUC. The AUC (min•mmHg/h) was calculated as time (minutes) spent with blood pressure below MAP_OPT_ and blood pressure deviation (mmHg) below MAP_OPT_, and then normalized for the duration of monitoring (hours) [10]. In addition, we calculated the percentage of time that neonates spent with blood pressure below the ga + 5 in each period. Finally, we analyzed the rSO_2_ using the mean between right and left cerebral hemispheres.

### Neurodevelopmental evaluation

When the children were 21–32 months of age, they were evaluated for neurodevelopmental function in a single visit at KKI during a routinely scheduled clinical visit or a one-time research visit. Clinical visits were part of routine and regularly scheduled care in the KKI NICU follow-up clinic and included a neurologic exam, administration of the Capute Scales [14, 15] completed by or under the supervision of a developmental pediatrician or neonatologist, and a motor evaluation by a physical therapist. The Capute Scales are designed to assess language and visual–motor streams of development in children with a cognitive age ≤36 months. At research visits, the children participated in a battery that included the Mullen Early Scales of Development and the Gross Motor Function Measure (GMFM) administered by a neuropsychologist or a neurodevelopmental pediatrician. The Mullen is a comprehensive standardized measure of visual perception, language, and motor skill acquisition in children from birth to 68 months of age [16]. The GMFM is a detailed and quantitative measure of gross motor development that is frequently used to evaluate motor skill acquisition in individuals with cerebral palsy [17]. Neurodevelopmental outcomes were classified as impaired based on a Mullen Early Learning Composite Standard Score or Capute Full Scale Developmental Quotient <85 and a Gross Motor Function Classification (GMFC) of II-V based on GMFM performance or clinical neurologic and motor exam [18, 19]. This classification translates functionally to below average cognitive ability and the ability to walk with limitations. In contrast, neurodevelopmental outcomes were classified as unimpaired based on a Mullen Early Learning Composite Standard Score or Capute Full Scale Developmental Quotient ≥ 85 and a GMFC of I based on GMFM performance or clinical neurologic and motor exam. This classification translates functionally to average cognitive ability and the ability to walk without limitations. Investigators who conducted or supervised the neurodevelopmental examinations (VJB, GG, EC) were blinded to the blood pressure and autoregulation data.

### Statistical analysis

Data were analyzed with SigmaPlot (v11.0, Systat Software Inc., Chicago, IL) and SAS v9.2 (SAS Institute Inc., Cary, NC). Graphs were generated with GraphPad Prism (v5.03, GraphPad Software Inc., La Jolla, CA). We present the data as means with standard deviations (SD) or medians with interquartile ranges (IQR) when appropriate. Differences were considered significant at *p* < 0.05. Neurodevelopmental outcomes were dichotomized into impaired or unimpaired, and the Mullen Early Learning Composite scores were analyzed as a continuous variable. MAP_OPT_ values and the percentage of time spent with blood pressure below MAP_OPT_ during each period (hypothermia, rewarming, and the first 6 h of normothermia) were compared by using Wilcoxon signed rank tests. Blood pressure, MAP_OPT,_ and rSO_2_ data with respect to neurodevelopmental outcomes were tested separately within each time period. MAP_OPT_; the percentage of time spent with blood pressure below, at, or above MAP_OPT_; the maximal blood pressure deviation below or above MAP_OPT_; AUC; the percentage of time spent with blood pressure below ga + 5; and rSO_2_ were compared between children with and without impairments by using Mann Whitney rank sum tests. MAP_OPT_, blood pressure data in relation to MAP_OPT_ and ga + 5, and rSO_2_ were compared to Mullen scores by using Spearman correlations. Seizure activity and the receipt of a vasopressor (dopamine, dobutamine, or epinephrine) were compared between children with and without impairments by using Fisher exact tests.

## Results

Twenty-eight neonates with HIE received therapeutic hypothermia and had HVx monitoring. Nineteen of those children participated in neurodevelopmental follow-up examinations at 21–32 months of age. Therefore, data are presented for 19 children (10 girls, 9 boys). Their mean gestational age was 38.9 weeks (*n =* 19; SD = 1.5). During the autoregulation monitoring period, 11 (58 %) neonates had clinical or electrographic seizures that were treated with phenobarbital. Four of these neonates received additional antiepileptic therapy, including levetiracetam, fosphenytoin, lorazepam, or topiramate for persistent seizures. Thirteen (68 %) neonates received vasopressors during HVx monitoring, including 13 with dopamine, four with dobutamine, and one with epinephrine. Morphine infusions were administered to four neonates, and a hydromorphone infusion was given to one neonate. Fourteen neonates were intubated for synchronized intermitted mandatory ventilation (13) or high frequency jet ventilation (1). Seven neonates received nasal continuous positive airway pressure or high-flow nasal cannula respiratory support. During the rewarming period, four intubated neonates had adjustments to their ventilator respiratory rate (range of increase in respiratory rate: 5–14 breaths/min) or peak inspiratory pressure (range of change in peak inspiratory pressure: 1–6 cmH_2_O). One neonate had inhaled nitric oxide initiated during rewarming. Nine neonates had adjustments made in the inhaled oxygen concentration delivered through nasal cannula (2), high flow nasal cannula (1), or endotracheal tube (6; range of change in inhaled oxygen concentration: 5–55 %). No patients received extracorporeal membrane oxygenation (ECMO). Clinical data upon admission to the NICU and physiologic and laboratory data are presented in Tables 1 and 2.

**Table 1:** Clinical characteristics of neonates with hypoxic-ischemic encephalopathy upon admission to the neonatal intensive care unit

Parameter	N	Median (IQR)
Apgar at 1 min	19	1 (1, 2)
Apgar at 5 min	19	3 (2, 5)
Apgar at 10 min	19	5 (3, 6)
Cord blood pH	15	7.00 (6.91, 7.05)
Cord blood base deficit	15	−11 (–10,–15)
Arterial pH within 1 h of life	19	7.07 (6.94, 7.24)
Base deficit within 1 h of life	19	−17 (–13,–22)

*IQR* interquartile range

**Table 2:** Physiologic and laboratory data during autoregulation monitoring

Parameter	Hypothermia (*n =* 19)	Rewarming (*n =* 17)	Normothermia (*n =* 16)
Temperature (°C)	33.5 (0.5)	35.1 (0.9)	36.8 (0.3)
Heart rate (bpm)	110 (17)	117 (17)	133 (18)
pH^a^	7.38 (0.05)	7.36 (0.07)	7.36 (0.06)
PaO_2_^a^	130 (72)	100 (42)	121 (50)
PaCO_2_^a^	43 (8)	50 (10)	48 (6)
Hemoglobin (g/dL)	15.7 (2.1)	14.0 (0.5)	13.5 (0.7)

All values are presented as mean (SD)

*Bpm* beats per minute

^a^Arterial blood gas

### Neurodevelopmental outcomes

Nineteen children had neurodevelopmental outcomes evaluated at a median age of 25 months (range, 21–32 months). Fifteen children were evaluated in research visits. Because one of those participants did not complete the GMFM, the GMFC level was determined by using the Mullen Gross Motor performance and clinical judgment. Four children had clinical evaluations. Children who participated in the full research battery had a mean performance on the Mullen Early Learning Composite within the normal range (*n =* 15; mean = 88.87; SD = 18.52). GMFM scores (*n =* 14; mean = 84.23; SD = 22.57) were similar to those of 2–4-year-old children with cerebral palsy who can walk without assistance (*n =* 25; mean = 81.2; SD = 13.5) [18]. The children who had clinical visits also had average mean performance (*n =* 4; mean = 86.00; SD = 51.61). Overall, 11 (58 %) children had typical performance or mild delays in neurodevelopment based on cognitive performance in the average range and the ability to walk without limitations. These 11 children were coded as having an unimpaired neurodevelopmental outcome for the analysis. Eight (42 %) children had more significant delays based on below-average cognitive performance or the inability to walk without limitations. These eight children were coded as having an impaired neurodevelopmental outcome for the analysis. Seizures or the receipt of a vasopressor were not associated with having an impaired neurodevelopmental outcome (*p* = 1.000 for seizures; *p* = 1.000 for vasopressors).

### Autoregulatory vasoreactivity

**Fig. 2:**
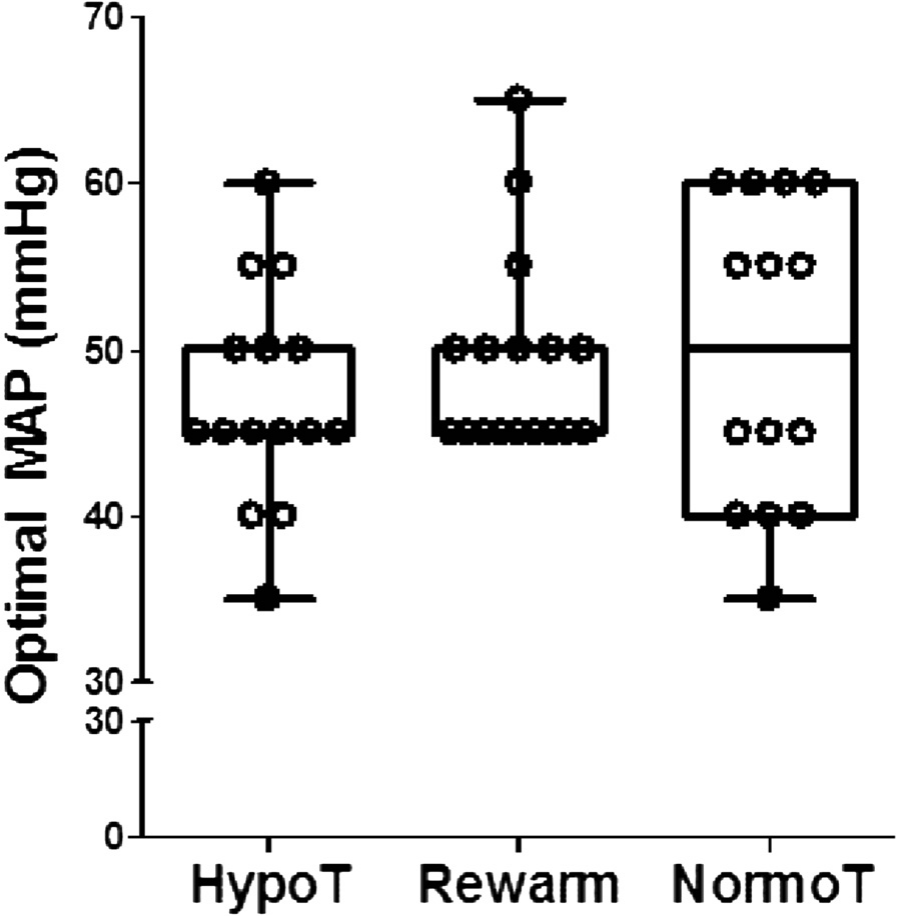
Optimal mean arterial blood pressure (MAP) values were similar during hypothermia (hypoT; *n =* 15), rewarming (rewarm; *n =* 17), and the first 6 h of normothermia (normoT; *n =* 14). *p* = 0.831 for hypothermia vs. rewarming; *p* = 0.313 for hypothermia vs. normothermia; *p* = 0.685 for rewarming vs. normothermia by Wilcoxon signed rank tests. Box plots with whiskers (5^th^–95^th^ percentiles) are shown. Each circle represents one neonate

All 19 neonates with measured neurodevelopmental outcomes were monitored during therapeutic hypothermia. HVx monitoring was terminated before rewarming in one neonate because of technical complications with monitoring and in a second who was transferred to the pediatric ICU for potential ECMO (ECMO was not initiated). One neonate did not receive HVx monitoring during normothermia because the arterial blood pressure cannula was removed. MAP_OPT_ was identified in 15/19 (79 %) neonates during hypothermia, 17/17 (100 %) during rewarming, and 14/16 (88 %) during the first 6 h of normothermia. HVx was monitored for a median of 30.5 h (IQR, 22.4–46.5) during hypothermia, 6.5 h (IQR, 5–8) during rewarming, and 6 h (IQR, 6–6) during normothermia. MAP_OPT_ ranged from 35 to 65 mmHg, with the majority of MAP_OPT_ values between 45 and 55 mmHg. Values for MAP_OPT_ were similar during hypothermia, rewarming, and the first 6 h of normothermia (*p* = 0.831 for hypothermia vs. rewarming; *p* = 0.313 for hypothermia vs. normothermia; and *p* = 0.685 for rewarming vs. normothermia; Fig. 2).

**Fig. 3:**
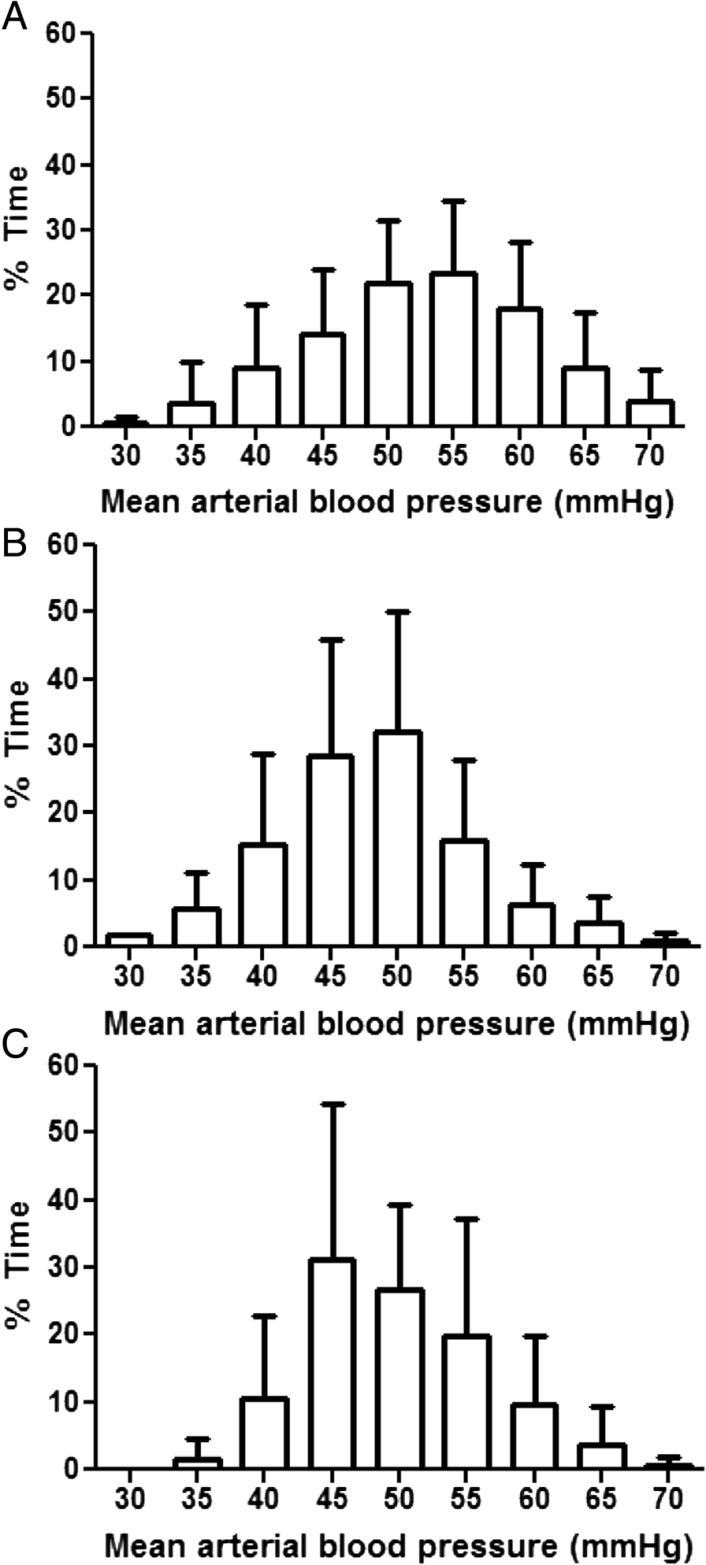
The percentages of time during hypothermia (*n =* 19; **a**), rewarming (*n =* 17; **b**), and normothermia (*n =* 16; **c**) that neonates spent at each level of mean arterial blood pressure. Data are shown as means with SDs

The MAP ranged from 30 to 70 mmHg but remained between 40 and 60 mmHg most of the time (Fig. 3). The percentage of time that neonates spent with blood pressure below MAP_OPT_ was similar between time periods. More specifically, neonates spent a median of 6 % (IQR, 1–25) of the hypothermia period and 41 % (IQR, 8–59) of the rewarming period with blood pressure below MAP_OPT_ (*p* = 0.119). Neonates spent a median of 31 % (IQR, 0–87 %) of the normothermia period with blood pressure below MAP_OPT_ (*p* = 0.083 for hypothermia vs. normothermia; *p* = 0.903 for rewarming vs. normothermia).

**Fig. 4:**
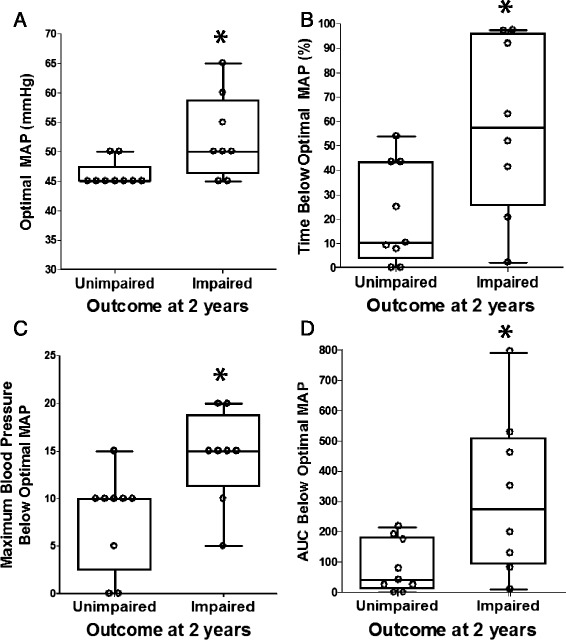
Optimal mean arterial blood pressure (MAP) and blood pressure below optimal MAP during the neonatal rewarming period in relation to neurodevelopmental outcome at approximately 2 years of age. In comparison to children without impairments (*n =* 9; unimpaired), children who developed impairments (*n =* 8; impaired) had higher optimal MAP values (*p = 0.023; **a**), spent a greater percentage of time with blood pressure below optimal MAP (*p = 0.048; **b**), had greater maximal blood pressure deviation below optimal MAP (*p = 0.019; **c**), and had greater area under the curve (AUC) below optimal MAP (*p = 0.039; **d**) during rewarming. Data were analyzed by Mann Whitney rank sum tests. Box plots with whiskers (5^th^–95^th^ percentiles) are shown. Each circle represents one child

Values for MAP_OPT_ during rewarming were significantly higher among children who developed impairments (*n =* 8) than in those who were unimpaired (*n =* 9; *p* = 0.023; Fig. 4a). MAP_OPT_ values were similar between children with impairments (*n =* 5) and those without impairments during hypothermia (*n =* 10; *p* = 0.949) and during normothermia (*n =* 7 children with impairments; *n =* 7 children without impairments; *p* = 0.383).

Neurodevelopmental outcome of children at approximately 2 years was associated with a longer duration of blood pressure below the individual neonate's MAP_OPT_ during the neonatal rewarming period. Children who developed impairments (*n =* 8) spent a greater percentage of time with blood pressure below MAP_OPT_ than did children without impairments (*n =* 9; *p* = 0.048; Fig. 4b). Additionally, children with impairments (*n =* 8) had greater maximal blood pressure deviation below MAP_OPT_ (*p* = 0.019; Fig. 4c) and greater AUC below MAP_OPT_ (*p* = 0.039; Fig. 4d) during rewarming than did those without impairments (*n =* 9). No associations were identified between impairment at 2 years and the percentage of time spent with blood pressure below MAP_OPT,_ maximal blood pressure deviation below MAP_OPT,_ or AUC during hypothermia and normothermia (*p* > 0.10 for all comparisons; Additional file 1: Table S1).

**Fig. 5:**
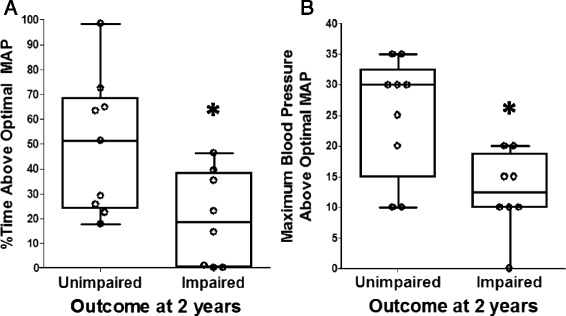
Blood pressure above the optimal mean arterial blood pressure (MAP) during the neonatal rewarming period in relation to neurodevelopmental outcome at approximately 2 years of age. When compared to children without impairments (*n =* 9; unimpaired), those who developed impairments (*n =* 8; impaired) spent a lower percentage of the rewarming period with blood pressure above optimal MAP (*p = 0.039; **a**) and had less maximal blood pressure deviation above optimal MAP (*p = 0.021; **b**) during rewarming. Data were analyzed by Mann Whitney rank sum tests. Box plots with whiskers (5^th^–95^th^ percentiles) are shown. Each circle represents one child

Better neurodevelopmental outcome was associated with greater time spent with blood pressure above MAP_OPT_ and greater blood pressure deviation above MAP_OPT_ during rewarming. Children who developed impairments (*n =* 8) spent a smaller percentage of the rewarming period with blood pressure above MAP_OPT_ (*p* = 0.039; Fig. 5a) and had less maximal blood pressure deviation above MAP_OPT_ (*p* = 0.021; Fig. 5b) than did children without impairments (*n =* 9). This association was not present for hypothermia or normothermia (*p* > 0.10 for all comparisons; Additional file 1: Table S1). Moreover, disability was not associated with the percentage of time spent with blood pressure at MAP_OPT_ in any time period (*p* > 0.10 for all comparisons; Additional file 1: Table S1).

**Fig. 6:**
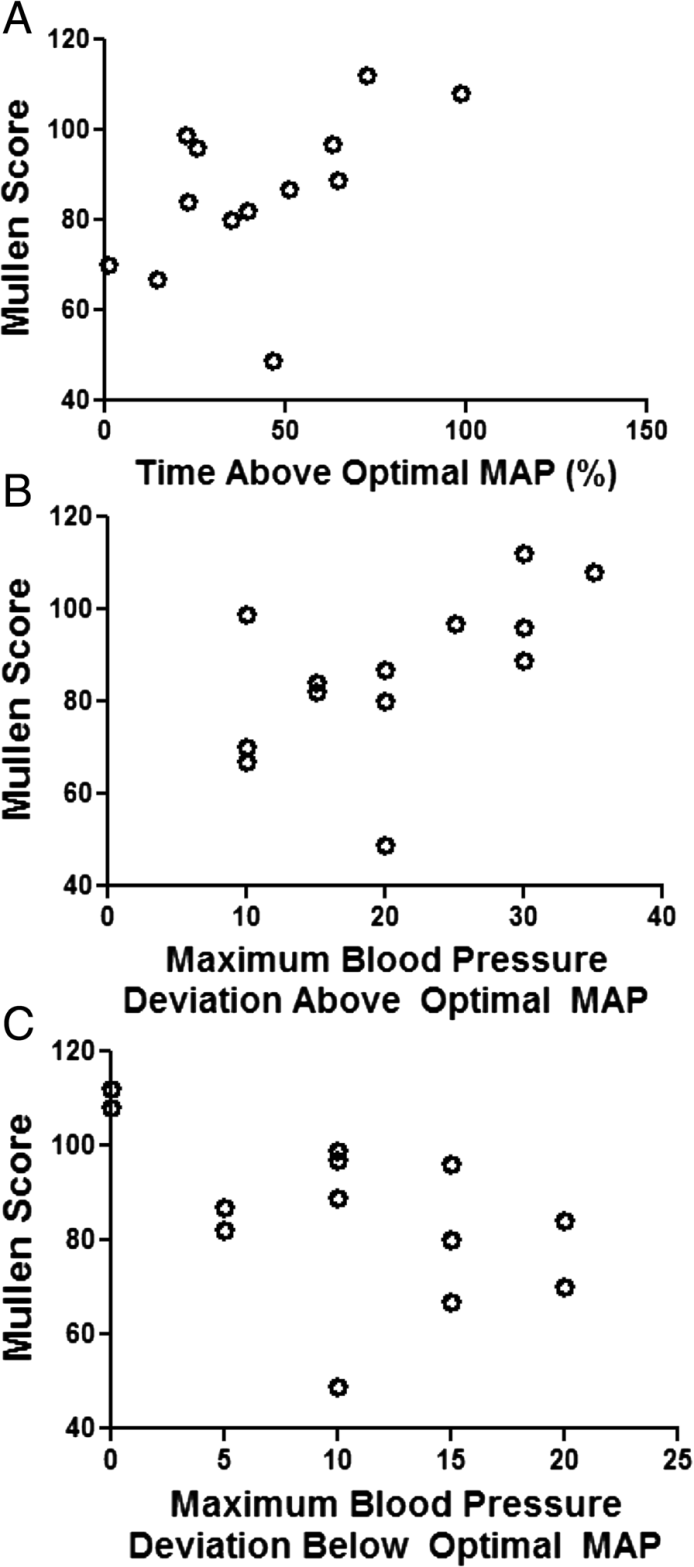
Blood pressure in relation to the optimal mean arterial blood pressure (MAP) during the neonatal rewarming period and the Mullen score at approximately 2 years of age. Higher Mullen scores correlated with a greater percentage of the rewarming period spent with blood pressure above optimal MAP (*n =* 13; r = 0.560, *p* = 0.044; **a**) and greater maximal blood pressure deviation above optimal MAP (*n =* 13; r = 0.585; *p* = 0.035; **b**). Lower Mullen scores correlated with greater maximal blood pressure deviation below optimal MAP (*n =* 13; r = –0.563; *p* = 0.044; **c**). Data were analyzed by Spearman correlations. Each circle represents one child

The Mullen score at the 2-year evaluation also correlated with blood pressure in relation to MAP_OPT_ during the neonatal rewarming period. A higher Mullen score correlated with a greater percentage of time spent in the rewarming period with blood pressure above MAP_OPT_ (*n =* 13; r = 0.560, *p* = 0.044; Fig. 6a) and a greater maximal blood pressure deviation above MAP_OPT_ during rewarming (*n =* 13; r = 0.585; *p* = 0.035; Fig. 6b). Similarly, maximal blood pressure deviation below MAP_OPT_ during rewarming and the Mullen score were negatively correlated (*n =* 13; r = –0.563; *p* = 0.044; Fig. 6c). The proportion of the rewarming period spent with blood pressure below MAP_OPT_ did not correlate with the Mullen score (*n =* 13; r = –0.465 *p* = 0.102). No correlations were identified between the Mullen score and duration of time with blood pressure above or below MAP_OPT_ or blood pressure deviation from MAP_OPT_ during hypothermia or normothermia (*p* > 0.10 for all comparisons; Additional file 2: Table S2). The Mullen score also did not correlate with the percentage of time spent at MAP_OPT_ or with the AUC below MAP_OPT_ in any time period (*p* > 0.05 for all comparisons; Additional file 2: Table S2).

### Cerebral oximetry and blood pressure in relation to gestational age

When all children were analyzed (including those with an unidentifiable MAP_OPT_), the mean rSO_2_ in any period (hypothermia, rewarming, or normothermia) was not associated with future impairment or Mullen score (*p* > 0.10 for all comparisons; Additional file 1: Tables S1 and Additional file 2: Table S2). The percentages of time during the hypothermia, rewarming, and normothermia periods that neonates spent with blood pressure below ga + 5 also were not associated with future impairment or Mullen score (*p* > 0.10 for all comparisons; Additional file 1: Table S1 and Additional file 2: Table S2). Moreover, neonates spent little time with blood pressure below their gestational age (Fig. 3).

## Discussion

Several findings relevant to the treatment of neonatal HIE are suggested by this observational pilot study. The range of MAP with optimized cerebrovascular autoregulatory vasoreactivity may be identified by using HVx. Further, deviation from MAP_OPT_ during rewarming was associated with outcome. Children with impairments at approximately 2 years of age had significantly higher MAP_OPT_ values during the neonatal rewarming period than did children without impairments. Neurodevelopmental impairment in children was associated with more time spent at blood pressure below MAP_OPT_, having greater maximal blood pressure deviation below MAP_OPT_, and having greater AUC below MAP_OPT_ during the rewarming period. Children without impairments spent more time with blood pressure above MAP_OPT_ and had greater blood pressure deviation above MAP_OPT_ during rewarming than did those with impairments. Furthermore, higher Mullen scores at 2 years significantly correlated with neonates spending more time with blood pressure above MAP_OPT_ and having greater blood pressure deviation above MAP_OPT_ during rewarming. An association was observed only between neurodevelopmental outcome and blood pressure in relation to MAP_OPT_ during rewarming; no association was apparent between outcome and blood pressure during hypothermia or normothermia. Finally, neither the rSO_2_ nor time spent with blood pressure below ga + 5 during hypothermia, rewarming, or normothermia was associated with neurodevelopmental outcome. Although a causal relationship between blood pressure autoregulation and neurodevelopmental outcomes cannot be determined in this small, observational pilot study, our findings reveal an association between better neurodevelopmental outcomes and having blood pressures that remain within or above MAP_OPT_ during rewarming. They further suggest that identifying each individual neonate’s MAP_OPT_ with HVx may serve as a better method than rSO_2_ alone or rules based on gestational age to select blood pressure goals.

Although the overall performance of the 19 children evaluated at 2 years was in the average range, the mean cognitive scores were lower than those in normative samples [3, 6], and 42 % of the children had impairments in cognitive or motor function. The large variability in performance of the children who had clinical evaluations was likely due to the small number of children in this group. Nine (32 %) children with HIE who received autoregulation monitoring in the NICU did not have neurodevelopmental outcome data available for this study. Nonetheless, the observed associations between neurodevelopmental outcomes and autoregulation in the children with available data carry important considerations for the hemodynamic management of neonatal HIE that deserve further study.

Cerebral NIRS is often used to monitor neonates with HIE during therapeutic hypothermia [9, 10, 20–23] because invasive neurologic monitoring is generally not feasible in such patients. The predictive value of cerebral oximetry in relation to neurologic outcomes remains unclear in HIE. For neonates with HIE who had selective head cooling, higher cerebral oximetry values during hypothermia were associated with worse outcomes, including death, cerebral palsy, or global delay [22]. In contrast, other studies report that cerebral oximetry cannot predict poor neurologic outcome at 7–10 days of life [21] or severe encephalopathy [23]. Numerous factors that affect cerebral oxygen supply and demand create variability in cerebral oximetry and make immediate interpretation of the readings difficult. These factors include the administration of sedative or anti-epileptic medications, seizures, changes in oxygen supply, hyper/hypoventilation, and fluctuations in hemoglobin levels. Moreover, the decrease in cerebral metabolic rate during therapeutic hypothermia and the subsequent increase in metabolism during rewarming are confounders. Calculating the cerebral tissue oxygen extraction may offer better correlation with brain injury than regional cerebral oximetry alone [20, 23]. Altered brain oxygen consumption that may be related to dysfunctional autoregulation [24] and regional differences in cerebral perfusion [25] have been described in preterm neonates and neonates with HIE or perinatal arterial ischemic strokes. Methods to assess autoregulation by correlating blood pressure with tissue oxygen levels or oxygen extraction measured by NIRS are being tested in neonates [26–28].

We used the autoregulation metric HVx, which incorporates measures of both oxygenated and deoxygenated hemoglobin. Therefore, HVx should be minimally affected by parameters that change tissue oxygen extraction or supply, including temperature and metabolic demand. This method may enable clinicians to develop an individualized approach for neonates with HIE by identifying and aiming for the MAP_OPT_ at which autoregulation is most functional.

Our ability to identify MAP_OPT_ in neonates by using HVx showed that MAP_OPT_ values vary among individuals. Children who developed impairments had significantly higher MAP_OPT_ during rewarming from hypothermia than did children who did not develop impairments. Intracranial hypertension raises the limits of blood pressure autoregulation [29]. It is possible that severely injured neonates are at risk of elevated intracranial pressure during rewarming [30], which could shift the blood pressure autoregulation curve to higher pressures and increase MAP_OPT_. Identifying MAP_OPT_ would be particularly critical in these neonates to clarify hemodynamic goals that support autoregulation.

We also found an association between neurodevelopmental impairment and blood pressure deviation from MAP_OPT_ during rewarming. Children with impairments spent more time with blood pressure below MAP_OPT_, had greater maximal blood pressure deviation below MAP_OPT_, and had greater AUC below MAP_OPT_ during rewarming than did those without impairments. Greater maximal blood pressure deviation below MAP_OPT_ correlated with a lower Mullen Early Learning Composite score. Likewise, having blood pressure that remained above MAP_OPT_ during rewarming was associated with less impairment. Children without impairments spent a greater proportion of the rewarming period with blood pressure above MAP_OPT_ and had greater blood pressure deviation above MAP_OPT_ than did children with impairments. Moreover, more time with blood pressure above MAP_OPT_ and greater maximal blood pressure deviation above MAP_OPT_ during rewarming correlated with a higher Mullen Early Learning Composite score.

There were no associations between the 2-year neurodevelopmental outcomes and blood pressure deviation from MAP_OPT_ during hypothermia or normothermia. The percentages of time that neonates spent with blood pressure below MAP_OPT_ were similar in the hypothermia, rewarming, and normothermia periods. Several possibilities might explain the association between blood pressure deviation from MAP_OPT_ during rewarming and neurodevelopmental outcomes. The inherent risk of secondary neuronal injury may be highest during rewarming. Additionally, severely injured neonates may have less stable cardiovascular regulation and diminished autoregulatory capacity during rewarming. We previously reported that spending more time and having greater blood pressure deviation below MAP_OPT_ during rewarming were associated with greater brain injury on MRI [9, 10]. This finding might be related to an increase in MAP_OPT_ during rewarming in severely injured neonates. Rewarming itself might adversely affect cerebral blood flow autoregulation and increase the risk of stroke [31]. Intracranial hypertension and hyperemia can occur in some brain-injured regions during rewarming [30]. Moreover, cytotoxicity from rewarming with resultant neuronal cell death may be enhanced in the post-hypoxic developing brain [32]. Other neural cells also are likely vulnerable to secondary injury after hypoxia in the developing brain. In the 24 h after rewarming, elevated serum glial fibrillary acidic protein, a biomarker of astrocyte injury, was associated with the greatest severity of clinical and MRI markers of brain injury in neonates with HIE [33].

Given the small sample size in this pilot study, we were unable to control for hemoglobin level, PaCO_2_, or sedation which might affect cerebral blood flow and potentially confound the interpretation of HVx. Neonates did not have any clinical changes during rewarming that would acutely change their hemoglobin level, such as blood transfusion or hemorrhage. Vasoreactivity is affected by changes in CO_2_ production [34], including those secondary to changing metabolic rate at different temperatures. Nonetheless, HVx is a useful metric during rewarming because it is derived from both oxygenated and deoxygenated hemoglobin and therefore should be minimally affected by shifts in the oxy-/deoxyhemoglobin balance with changing temperature and metabolic rate [9, 10, 13].

The effects of changing ventilatory support during rewarming on HVx are unclear. Changes in intrathoracic pressure can affect cerebral perfusion pressure [35] and oscillations in arterial oxygen levels from ventilator maneuvers are transmitted to the cerebral microcirculation [36]. Four intubated neonates in this study had adjustments in their ventilator respiratory rate and/or peak inspiratory pressure during rewarming. Because ventilator frequencies are filtered out before calculating HVx, [8] mechanical effects of ventilation should be minimal on HVx unless they substantially increase steady state cerebral venous blood volume. Changes in the inhaled oxygen concentration should also minimally affect HVx, which is derived from the amount of total cerebral hemoglobin and not just the oxyhemoglobin component. On the other hand, it is conceivable that the one neonate who received inhaled nitric oxide during rewarming had increased cerebral delivery of nitrite, which then could have produced cerebral vasodilation. Formal studies to examine the effects of changing ventilator support and oxygen supply on HVx measurements are warranted.

Regional SO_2_ and blood pressure based on ga + 5 were not associated with neurodevelopmental outcomes in this study. The “normal” MAP for a neonate is often assumed to be the neonate’s ga + 5 [11], a value that frequently serves as the goal blood pressure for critically ill neonates. Our findings in this pilot study suggest that using autoregulation monitoring to identify hemodynamic goals that support autoregulation may be superior to rSO_2_ or rules based on gestational age.

While the data suggest that maintaining a patient’s blood pressure near MAP_OPT_ might improve outcome, a cause and effect relationship between blood pressure autoregulation and neurodevelopmental outcome cannot yet be determined in this observational pilot study. The risks of raising a neonate’s blood pressure must be considered. While targeting the optimal cerebral perfusion pressure to support autoregulation has not yet been explored in neonates with HIE, it is being evaluated in adult traumatic brain injury [37].

Given the small sample size of this pilot study, we were unable to control for potential confounders such as gender, socioeconomic status and access to therapy services that might affect neurodevelopmental outcome. There are factors in addition to hemodynamic management that may correlate with neurodevelopmental outcomes in HIE, including abnormal EEG and brain imaging [38] and non-neurologic co-morbidities. Also, the type of neonatal cerebral oximetry probe may affect the cerebral oximetry measurements in neonates [39]. Because HVx monitoring could only begin after an arterial blood pressure cannula was established during hypothermia, we analyzed the data as the percentage of the autoregulation monitoring period and normalized the AUC for the duration of monitoring to account for different durations of monitoring during the hypothermia period. Early instability in cerebral autoregulation immediately after the perinatal event was not captured in this study. We were able to monitor HVx more consistently during rewarming and the first 6 h of normothermia. HVx measures only the regional frontal cortex and cannot be used to assess other regions of the brain, including those thought to be most at risk from neonatal hypoxic ischemia [40]. Variability in regional brain oxygenation has been reported in models of neonatal HIE, including differences in oxygenation between cortical and thalamic regions [41]. Additionally, we could not validate HVx against other measures of cerebral blood flow, such as transcranial Doppler, because continuous Doppler is not feasible for 3–4 days in neonates. Nonetheless, HVx has been validated against laser-Doppler in a swine model of HIE [13], HVx correlates with intracranial pressure-derived autoregulation measures in patients [42], and HVx has been validated against transcranial Doppler in identifying MAP_OPT_ during cardiopulmonary bypass [43].

## Conclusions

In this observational pilot study of neonatal HIE and therapeutic hypothermia, we used HVx monitoring to identify associations between cerebrovascular autoregulatory vasoreactivity during the neonatal rewarming period and 2-year neurodevelopmental outcomes. MAP_OPT_ varied among individual neonates and was higher during rewarming in children who developed impairments than in those who did not. Blood pressure deviation below MAP_OPT_ during rewarming was associated with greater impairment and lower cognitive scores. Although future studies are warranted, our pilot data suggest that individualizing blood pressure goals based on MAP_OPT_, especially during the rewarming period, may be superior to rSO_2_ alone or blood pressure goals based on gestational age to support autoregulation and improve neurodevelopmental outcomes.

## Additional files

Additional file 1: Table S1. Blood pressure data in relation to the optimal mean arterial blood pressure or gestational age, regional cerebral oxygen saturation, and neurodevelopmental disability. (DOCX 19 kb) Additional file 2: Table S2. Blood pressure in relation to the optimal mean arterial blood pressure and the Mullen score. (DOC 49 kb)

## Abbreviations

HIE: Hypoxic-ischemic encephalopathy
HVx: Hemoglobin volume index
MAP_OPT_: Mean arterial blood pressure at which autoregulatory vasoreactivity is optimal
rSO_2_: Regional cerebral oximetry
NIRS: Near-infrared spectroscopy
rTHb: Relative total tissue hemoglobin
NICU: Neonatal intensive care unit
EEG: Electroencephalogram
MRI: Magnetic resonance imaging
AUC: Area under the curve
GMFM: Gross motor function measure
GMFC: Gross motor function classification
ECMO: Extracorporeal membrane oxygenation
PaCO_2_: Partial pressure of carbon dioxide in blood
