# Supplementary material for: A pilot cohort study of cerebral autoregulation and 2-year neurodevelopmental outcomes in neonates with hypoxic-ischemic encephalopathy who received therapeutic hypothermia
Source: BMC Neurol. 2015 Oct 20;15:209. doi: 10.1186/s12883-015-0464-4 (PMC4618147; doi:10.1186/s12883-015-0464-4)
Supplement: Additional file 1: Table S1. — Blood pressure data in relation to the optimal mean arterial blood pressure or gestational age, regional cerebral oxygen saturation, and neurodevelopmental disability. (DOCX 19 kb) [file 12883_2015_464_MOESM1_ESM.docx]

**Additional file 1 Table S1.** Blood pressure data in relation to the optimal mean arterial blood pressure or gestational age, regional cerebral oxygen saturation, and neurodevelopmental disability

| **Percentage of time spent with blood pressure below optimal MAP** | | | | |
| --- | --- | --- | --- | --- |
| **Period** | **Disability** | **N** | **Median (IQR) (%)** | ***p*-value** |
| Hypothermia | No/mild | 10 | 5 (1, 29) | 0.759 |
|  | Moderate/severe | 5 | 16 (1, 43) |  |
| Rewarming | No/mild | 9 | 10 (4, 44) | 0.048 |
|  | Moderate/severe | 8 | 58 (26, 96) |  |
| Normothermia | No/mild | 7 | 59 (0, 86) | 0.710 |
|  | Moderate/severe | 7 | 2 (0, 87) |  |
|  |  |  |  |  |
| **Percentage of time spent with blood pressure at optimal MAP** | | | | |
| **Period** | **Disability** | **N** | **Median (IQR) (%)** | ***p*-value** |
| Hypothermia | No/mild | 10 | 19 (9, 30) | 0.951 |
|  | Moderate/severe | 5 | 24 (3, 31) |  |
| Rewarming | No/mild | 9 | 28 (25, 35) | 0.470 |
|  | Moderate/severe | 8 | 14 (4, 41) |  |
| Normothermia | No/mild | 7 | 14 (8, 31) | 0.620 |
|  | Moderate/severe | 7 | 10 (2, 33) |  |
|  |  |  |  |  |
| **Percentage of time spent with blood pressure above optimal MAP** | | | | |
| **Period** | **Disability** | **N** | **Median (IQR) (%)** | ***p*-value** |
| Hypothermia | No/mild | 10 | 77 (36, 89) | 0.951 |
|  | Moderate/severe | 5 | 57 (28, 97) |  |
| Rewarming | No/mild | 9 | 51 (24, 69) | 0.039 |
|  | Moderate/severe | 8 | 19 (0.4, 38) |  |
| Normothermia | No/mild | 7 | 10 (1, 62) | 0.383 |
|  | Moderate/severe | 7 | 67 (3, 97) |  |
|  |  |  |  |  |
| **Maximal blood pressure deviation below optimal MAP** | | | | |
| **Period** | **Disability** | **N** | **Median (IQR) (%)** | ***p*-value** |
| Hypothermia | No/mild | 10 | 10 (5, 15) | 0.454 |
|  | Moderate/severe | 5 | 15 (5, 20) |  |
| Rewarming | No/mild | 9 | 10 (5, 10) | 0.019 |
|  | Moderate/severe | 8 | 15 (10, 20) |  |
| Normothermia | No/mild | 7 | 20 (0, 20) | 0.456 |
|  | Moderate/severe | 7 | 5 (0, 20) |  |
|  |  |  |  |  |
| **Maximal blood pressure deviation above optimal MAP** | | | | |
| **Period** | **Disability** | **N** | **Median (IQR) (%)** | ***p*-value** |
| Hypothermia | No/mild | 10 | 30 (20, 35) | 0.900 |
|  | Moderate/severe | 5 | 25 (25, 35) |  |
| Rewarming | No/mild | 9 | 30 (15, 35) | 0.021 |
|  | Moderate/severe | 8 | 15 (10, 20) |  |
| Normothermia | No/mild | 7 | 15 (5, 30) | 1.000 |
|  | Moderate/severe | 7 | 20 (15, 25) |  |
|  |  |  |  |  |
| **AUC below optimal MAP** | | | | |
| **Period** | **Disability** | **N** | **Median (IQR) (**min•mmHg/h**)** | ***p*-value** |
| Hypothermia | No/mild | 10 | 19 (3, 107) | 0.854 |
|  | Moderate/severe | 5 | 55 (2, 270) |  |
| Rewarming | No/mild | 9 | 41 (23, 183) | 0.039 |
|  | Moderate/severe | 8 | 274 (94, 509) |  |
| Normothermia | No/mild | 7 | 229 (0, 483) | 0.456 |
|  | Moderate/severe | 7 | 6 (0, 459) |  |
|  |  |  |  |  |
| **Regional cerebral oxygen saturation** | | | | |
| **Period** | **Disability** | **N** | **Median (IQR) (**%**)** | ***p*-value** |
| Hypothermia | No/mild | 11 | 83 (80, 91) | 0.433 |
|  | Moderate/severe | 8 | 88 (82, 92) |  |
| Rewarming | No/mild | 9 | 87 (79, 91) | 0.194 |
|  | Moderate/severe | 8 | 91 (82, 94) |  |
| Normothermia | No/mild | 9 | 85 (79, 91) | 0.204 |
|  | Moderate/severe | 7 | 91 (89, 93) |  |
|  |  |  |  |  |
| **Percentage of time spent with blood pressure below gestational age + 5** | | | | |
| **Period** | **Disability** | **N** | **Median (IQR) (**%**)** | ***p*-value** |
| Hypothermia | No/mild | 11 | 11 (2, 20) | 0.772 |
|  | Moderate/severe | 8 | 11 (3, 20) |  |
| Rewarming | No/mild | 9 | 10 (4, 44) | 0.962 |
|  | Moderate/severe | 8 | 15 (2, 34) |  |
| Normothermia | No/mild | 9 | 3 (1, 38) | 0.958 |
|  | Moderate/severe | 7 | 5 (0.3, 33) |  |

MAP, mean arterial blood pressure; AUC, area under the curve.
