# Supplementary material for: A pilot cohort study of cerebral autoregulation and 2-year neurodevelopmental outcomes in neonates with hypoxic-ischemic encephalopathy who received therapeutic hypothermia
Source: BMC Neurol. 2015 Oct 20;15:209. doi: 10.1186/s12883-015-0464-4 (PMC4618147; doi:10.1186/s12883-015-0464-4)
Supplement: Additional file 2: Table S2. — Blood pressure in relation to the optimal mean arterial blood pressure and the Mullen score. (DOC 49 kb) [file 12883_2015_464_MOESM2_ESM.doc]

**Additional file 2 Table S2.** Blood pressure in relation to the optimal mean arterial blood pressure and the Mullen score.

| **Percentage of time spent with blood pressure below optimal MAP** | | | |
| --- | --- | --- | --- |
| **Period** | **N** | **R** | ***p*-value** |
| Hypothermia | 11 | –0.241 | 0.450 |
| Rewarming | 13 | –0.465 | 0.102 |
| Normothermia | 11 | 0.211 | 0.520 |
|  |  |  |  |
| **Percentage of time spent with blood pressure at optimal MAP** | | | |
| **Period** | **N** | **R** | ***p*-value** |
| Hypothermia | 11 | –0.173 | 0.595 |
| Rewarming | 13 | –0.264 | 0.372 |
| Normothermia | 11 | 0.518 | 0.095 |
|  |  |  |  |
| **Percentage of time spent with blood pressure above optimal MAP** | | | |
| **Period** | **N** | **R** | ***p*-value** |
| Hypothermia | 11 | 0.345 | 0.283 |
| Rewarming | 13 | 0.560 | 0.044 |
| Normothermia | 11 | –0.436 | 0.168 |
|  |  |  |  |
| **Maximal blood pressure deviation below optimal MAP** | | | |
| **Period** | **N** | **R** | ***p*-value** |
| Hypothermia | 11 | –0.314 | 0.324 |
| Rewarming | 13 | –0.563 | 0.044 |
| Normothermia | 11 | 0.227 | 0.484 |
|  |  |  |  |
| **Maximal blood pressure deviation above optimal MAP** | | | |
| **Period** | **N** | **R** | ***p*-value** |
| Hypothermia | 11 | 0.295 | 0.369 |
| Rewarming | 13 | 0.585 | 0.035 |
| Normothermia | 11 | –0.347 | 0.283 |
|  |  |  |  |
| **AUC below optimal MAP** | | | |
| **Period** | **N** | **R** | ***p*-value** |
| Hypothermia | 11 | –0.255 | 0.433 |
| Rewarming | 13 | –0.511 | 0.070 |
| Normothermia | 11 | 0.360 | 0.257 |
|  |  |  |  |
| **Regional cerebral oxygen saturation** | | | |
| **Period** | **N** | **R** | ***p*-value** |
| Hypothermia | 15 | 0.257 | 0.346 |
| Rewarming | 13 | –0.099 | 0.737 |
| Normothermia | 12 | –0.105 | 0.733 |
|  |  |  |  |
| **Percentage of time spent with blood pressure below gestational age + 5** | | | |
| **Period** | **N** | **R** | ***p*-value** |
| Hypothermia | 15 | –0.029 | 0.913 |
| Rewarming | 13 | –0.223 | 0.447 |
| Normothermia | 12 | –0.004 | 0.974 |

MAP, mean arterial blood pressure; AUC, area under the curve.
